# Women’s status, empowerment, and utilization of skilled delivery services in Papua New Guinea: an empirical analysis based on structural equation modeling

**DOI:** 10.3389/fpubh.2023.1192966

**Published:** 2024-01-09

**Authors:** Hao Shen, Hang Zhao, Baoqin Wang, Yi Jiang

**Affiliations:** ^1^School of Public Health, Chongqing Medical University, Chongqing, China; ^2^Research Center for Medical and Social Development, Chongqing Medical University, Chongqing, China

**Keywords:** Papua New Guinea, women’s empowerment, status of women, skilled birth attendants, structural equation modeling

## Abstract

**Background:**

Skilled birth attendants (SBA) facilitate identifying and overcoming labor problems and saving lives. With one of the highest maternal death rates in the Asia-Pacific area, SBA utilization during childbirth among Papua New Guinea (PNG) women remains low. Women’s status and empowerment are important factors in maternal and child health services and critical to maternal and child health development. This study is intended to apply structural equation modeling based on data from the Demographic and Health Survey (DHS) to evaluate the causal relationship between women’s status, empowerment, and SBA utilization in PNG and the mechanisms of their influence.

**Methods:**

This study employed data from the 2016–2018 Papua New Guinea Demographic Health Survey (PNG DHS), which recruited 18,175 women aged 15–49 years. A multi-stage sample and a structured questionnaire were used to collect information on maternal health, women’s empowerment, and related topics. STATA 17.0 was used to describe the data, while MPLUS 8.2 was employed for structural equation modeling and pathway analysis.

**Results:**

The two empowerment dimensions of household decision-making (standardized path coefficient, *β* = 0.049, *p* < 0.05) and access to health services (*β* = 0.069, *p* < 0.01) were positively associated with SBA utilization, while the association between attitudes toward partner violence and SBA utilization was not statistically significant. In addition, mediation analysis revealed that education indirectly influenced SBA utilization through access to health services (*β* = 0.011, 95% CI: 0.002, 0.022).

**Conclusion:**

The findings confirmed the direct and indirect effects of women’s status and empowerment on SBA utilization in PNG. Therefore, a call for further evidence-based interventions in PNG and possibly Pacific Small Island Developing States (PSIDS) is needed to improve women’s educational attainment, household decision-making, and access to health services to enhance maternal and newborn health and well-being.

## 1 Introduction

According to Sustainable Development Goal 3.1 (SDG-3.1), the maternal mortality ratio (MMR) is a global public health issue (1), with approximately 287,000 maternal deaths worldwide in 2020 and 41.8% occurring in least developed countries (2). Papua New Guinea (PNG) is the largest Pacific Small Island Developing State (PSIDS) (3), having one of the highest MMRs in the Asia-Pacific region (4), still faces significant obstacles in achieving SDG-3.1. Most PNG maternal deaths are caused by postpartum hemorrhage, sepsis, embolism, and other complications resulting from pregnancy or delivery (5).

Skilled birth attendants (SBAs) can effectively overcome problems during labor (6, 7), thereby considerably reducing maternal and neonatal mortality and contributing to the SDG-3.1 and SDG-3.2 goals (1). However, only half (56.5%) of women in PNG have SBA during childbirth (8), significantly lower than the average SBA utilization in the Western Pacific region (9). Thus, there is a need to explore possible pathways affecting SBA utilization to improve maternal and child health.

Prior studies have investigated supply-side challenges to SBA consumption among PNG women, such as recurring shortages of primary healthcare resources (e.g., human, material, and financial) and ineffective or inefficient primary healthcare systems (10–14). Demand-side barriers, such as socio-economic factors (e.g., poverty and low education levels), cultural factors (e.g., beliefs and customs that favor traditional delivery), and geographic factors (e.g., physical distance and lack of transportation) also affect the demand, access, and utilization of SBA among PNG women (15–17).

In addition, gender inequality is one of the significant barriers to SBA utilization among women in PNG that severely hinders the continued development of maternal and child health (18). PNG is the most gender-inequitable country globally, with a Gender Inequity Index (GII) ranking of 169/170 in 2021 (19). Some studies indicated that gender inequality in PNG stems from the entrenched patriarchy in society and the perpetuation of gender-based subversive violence to sustain patriarchy and further gender inequality (20–22), resulting in the low status of women and the denial of human rights and essential health services (23, 24). Related global research and policy reports indicated that strategies to eliminate gender inequality must involve efforts to improve the status of women and empower them to create an enabling environment for women to protect their health within patriarchal systems (25, 26).

Women’s status, or social status, is equivalent to “women’s recognized social position within a society’s hierarchy,” profoundly influenced by socio-economic, cultural, political, and other structural contexts (27, 28). Women’s educational attainment is the most prevalent indicator of their social standing (29). Education facilitates women’s understanding of their rights and maternal health services (30), including utilizing SBA (31). Improving women’s education is essential for achieving public health in low- and middle-income countries (LIMCS) (32).

Women’s empowerment is typically defined as “the process by which those who have been denied the ability to make strategic life choices acquire such an ability (33).” Extensive empirical research shows that women’s status and empowerment in developing countries positively impact SBA utilization (29, 31, 34–38). However, empirical research on the Pacific is more limited than in regions such as Asia and Africa. Meanwhile, the definition, conceptualization, and measurement of women’s empowerment are often different and controversial in other studies due to each country’s various socio-economic and cultural contexts and the fact that women’s empowerment is a complex underlying structure whose internal causal processes are not yet transparent (39). For example, on the dimension of women’s empowerment, the most relevant studies applying Demographic and Health Survey (DHS) data included attitudes toward partner violence and household decision-making to measure the impact of women’s empowerment on SBA utilization (29, 34–38). Some additionally incorporated elements like access to health services, gender-based negotiation, and social independence, which may often be independently valid in different contexts (29, 34–38), were also considered. Moreover, although the multidimensional structure of women’s empowerment has received increasing attention in terms of measurement methods (40), the majority of studies used summary measures or composite indices, which make it difficult to comprehend the contribution of each item to the dimensions and to explain the direction and patterns of change in women’s empowerment. In addition (34–36), few scholars have explored the mechanisms of action and potential pathways between the status of women, empowerment, and SBA utilization based on factor analysis and structural equation modeling (SEM).

Consequently, the purpose of this study was to use a nationally representative dataset of PNG to apply exploratory factor analysis (EFA) and confirmatory factor analysis (CFA) to propose and validate potential structures of women’s empowerment (household decision-making, attitudes toward partner violence, and access to health services). Furthermore, SEM was used to assess and test the causal relationship between women’s status, empowerment, and SBA utilization and its effective mechanism of action to improve the practical experience for enhancing women’s SBA employment.

### 1.1 Conceptual framework

**Figure 1:**
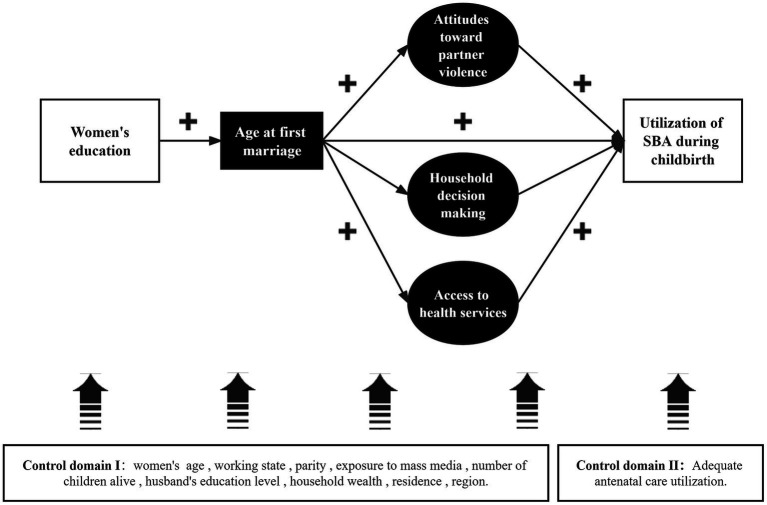
Predicted pathways for the impact of women’s status and multidimensional empowerment on SBA use.

This study is based on a comprehensive conceptual framework of “Gender Stratification Theory” to determine the social determinants of women’s underuse of reproductive health services in PNG (41). Figure 1 depicts the advances in women’s status as measured by education and feedback on women’s empowerment, which in turn increase women’s adoption of the SBA. Specifically, the theory and framework emphasize that women with more power will have more freedom to act independently and have control over their lives (e.g., household decision-making and access to health services).

The age of the first marriage mediates the path between education and SBA use. Three potential agent dimensions refer to the predictive path proposed by Shimamoto et al. (38). Specifically, a woman with a higher level of education tends to marry later (42–44), has more decision-making power in the household (45), has better access to health services (46) (which may also reflect the freedom of physical mobility) (47), and is better able to cope with domestic violence (48). In empirical studies of women’s empowerment employing DHS data, these three potential proxy dimensions are utilized more frequently. In contrast, this study also considers the sociodemographic characteristics of women and families as potential confounders and whether women have received effective antenatal care (ANC) services (49).

## 2 Methods

### 2.1 Data sources

This study employed cross-sectional data from the 2016–2018 Papua New Guinea Demographic and Health Survey (PNG DHS) to validate the association between women’s status, empowerment, and SBA use. The PNG DHS is a national survey of PNG residents aged 15–49 years in PNG. One of its objectives is to give updated information on the current primary population and health indicators, including maternal health, women’s empowerment, and demographic characteristics.

### 2.2 Sample design

The PNG DHS used the 2011 PNG National Population and Housing Census (NPHC) census units (CUs) as the sampling frame. The 22 provinces of PNG are divided into 43 sampling strata that distinguish between urban and rural differences, with no rural sampling strata in the Capital District. Each stratum was sampled using a two-stage stratified sampling method to obtain the sample. The first stage involved the selection of 800 CUs using probabilities proportional to size technique; the second stage used equal probability systematic sampling to select 24 households from each cluster, resulting in a final sample of approximately 19,200 households.

### 2.3 Study participants and sample size

The PNG DHS interviewed all eligible women (aged 15–49) in the family who were permanent residents of the family or visitors and had stayed at home the night before the survey. 18,175 women were identified for individual interviews, of which 15,198 completed the interview, and the response rate was 84%.

The study sample was restricted to married/cohabiting women who reported giving birth within the past 5 years. As traditional empowerment indicators focus more on the marital context (33), data on empowerment variables (e.g., women’s participation in decision-making) were collected only for married/cohabiting women. The sample of unmarried women was excluded, giving a total of 5,358 complete cases (weighted) after excluding outliers and missing values.

### 2.4 Data collection tools and techniques

The PNG DHS collected data using three structured questionnaires: household, women, and men. The questionnaires were modified from the standard Demographic and Health Survey Phase 7 questionnaire (DHS-7) to adequately represent PNG DHS-related questions and were pretested by trained enumerators (8).

### 2.5 Analysis strategies and measures

This research implemented SEM with latent variables, which are composed of fundamental structural and measurement models. In this study, the measurement model defines the link between three latent variables (i.e., household decision-making, attitudes toward partner violence, and access to health services) and their respective observable variables (indicators of individual empowerment). The structural model section describes the associations between the above latent variables. Five endogenous variables (whose cause is external to the model) and several exogenous variables are included (caused by one or more variables within the model). Endogenous variables include SBA utilization, household decision-making power, attitudes toward partner violence, access to health services, and age at first marriage. Exogenous variables included the sociodemographic characteristics of women and families and the efficiency of antenatal service utilization.

### 2.6 Endogenous variables

SBA utilization during childbirth was used as a dichotomous variable. Based on the 2016–2018 PNG DHS final report, 1 represented the use of SBA during childbirth, i.e., the respondent was assisted by a doctor/midwife/nurse (including trained community health workers)/trained village health volunteer at their last delivery, and 0 represented SBA was not used during childbirth, i.e., the previous delivery was assisted by a village birth attendant, family member, friend, or no one.

The DHS calculated age at first marriage as a continuous variable based on the century-month code between the respondent’s birth and first marriage/cohabitation.

Household decision-making was measured as a latent variable through three variables, i.e., who decides on the following: (1) the respondent’s health care; (2) large household purchases; and (3) visits to family/relatives. These three variables were recoded as dichotomous, with the joint/respondent decision alone being recoded as 1 and husband/partner/other person decision alone as 0.

As a latent variable, attitudes toward partner violence were measured using five variables, namely whether a respondent considered it to be justifiable for a wife to be beaten when she (1) went out without permission; (2) neglected her children; (3) argued with her husband; (4) refused to have sex with her husband; and (5) burned food. These five variables were recoded as dichotomous, with respondents’ perception of injustice recoded as 1 and justice do not know as 0.

Access to health services was measured as a latent variable by three variables, i.e., whether the respondent had difficulty in accessing health care in terms of (1) the money needed for treatment, (2) the distance to the health facility, (3) the permission to visit the health facility, and (4) going to the health facility alone. These four variables were recoded as dichotomous variables, with respondents perceiving no difficulties being recoded as 1 and significant difficulties as 0.

### 2.7 Exogenous variables

Based on the availability of data and the findings of previous PNG studies (15), 11 exogenous variables were included in this study, of which women’s education level was expressed as a continuous variable to reflect their highest years of education. Adequate antenatal care utilization was defined as a dichotomous variable according to the WHO (12), indicating whether the maternal participation in four or more antenatal visits, with 1 described as “yes” and 0 as “no.” The remaining variables were sociodemographic characteristics of women and households and were considered covariates.

The sociodemographic characteristics of women included the following variables: women’s age, parity, and number of children alive, which are included in the model as continuous variables. Work status is a dichotomous variable indicating whether women have been working in cash or kind for the last 12 months, with 1 defined as “employed” and 0 as “unemployed/unpaid employment.” Exposure to mass media is a dichotomous variable reflecting whether women have been exposed to at least one of reading newspapers, watching television, and listening to the radio, with 1 defined as “yes” and 0 as “no.”

The sociodemographic characteristics of households include the following variables: As a multi-categorical variable, the DHS constructed a composite index using principal components analysis based on the household’s consumer goods and housing characteristics. This index forms the corresponding household wealth quintile, which is defined as “poorest,” “poorer,” “middle,” “richer,” and “richest” from 0 to 4, respectively. Place of residence as a dichotomous variable, defined as 1 for urban and 0 for rural; region as a multi-categorical variable, defined as 0–3 for “Highland Region,” “Islands Region,” “Mormes Region,” and “Southern Region.”

### 2.8 Model analysis and steps

The study analysis was divided into three steps: first, descriptive analyses were conducted using STATA statistical software (version 17.0) on the full sample, where categorical variables were provided as percentages and standard errors, whereas continuous variables were presented as means and standard errors.

Second, Bartlett’s spherical and Kaiser-Meyer-Olkin (KMO) tests were performed using STATA statistical software. After the test results supported further factor analysis, the sample was randomly halved using the “splitsample” command, as recommended by relevant studies (50).

The data were then transferred to Mplus statistical software (version 8.3), where robust weighted least squares (WLSMV) estimates were applied to the two samples for exploratory factor analysis (EFA) and confirmatory factor analysis (CFA), respectively. The WLSMV estimation is appropriate for handling non-normal and categorical data (51). The EFA section was applied with a GEOMIN oblique rotation. The number of retained domains was determined by the scree plot and Kaiser’s criterion (retention of principal components with eigenvalues ≥1) (52). Items with small factor loadings (< | 0.3 |) and cross-loading were excluded, and Cronbach’s alpha coefficient was used as a measure of internal consistency (53). The CFA section validated the consistency of structure by the statistical significance of the model fit indices and unstandardized path coefficients, with the model fit indices selected to be more applicable to large samples, root mean square error of approximation (RMSEA) for categorical data, comparative fit indices (CFI), standardized root mean square residuals (SRMR), and Tucker- Lewis Index (TLI) (54).

Finally, the WLSMV estimator linked to the Probit function was applied in the Mplus statistical software (version 8.3) to estimate the model fit index, the statistical significance of the standardized path coefficients, the magnitude of the effect, and its direction for SEM. SEM can effectively control for measurement error to obtain estimates superior to those of regression analysis. The bootstrapping product coefficient method with 500 draws was applied to test the significance of indirect effects. Mutual covariation between exogenous variables is recognized because of the probability of their correlation. Conversely, mutual covariation between error terms of the assigned dimensions is enabled due to the possibility of correlation between unobserved components of the underlying structure. The analyses utilized complicated sampling procedures to account for individual weights, clusters, and sampling strata to get nationally representative PNG data.

## 3 Results

### 3.1 Descriptive analysis results

According to the descriptive statistics in Table 1, 57.82% of women used the SBA during their last birth, while 51.42% participated in ANC services four times or more. Regarding women’s empowerment, the mean scores of their family decision-making power and attitudes toward partner violence were in the upper middle range.

**Table 1:** Characteristics of married women who participated and had at least one birth in the last 5 years (*n* = 5,224 unweighted; *n* = 5,358 weighted), PNG DHS 2016–2018.

Variables	Mean/Percentage (%)	SE
**Endogenous variables**		
SBA utilization during childbirth		
No	42.18	1.83
Yes	57.82	1.83
Household decision-making(Mean, scored 0–3)	2.36	0.03
Attitudes toward partner violence(Mean, scored 0–5)	2.50	0.05
Access to health services(Mean, scored 0–4)	1.97	0.06
Age at first marriage	19.55	0.11
Exogenous variables		
Women’s education level	5.40	0.17
Age	30.04	0.14
Working state		
Unemployed/unpaid employment	83.47	1.35
Paid employment	16.53	1.35
Exposure to mass media		
No	53.14	1.87
Yes	46.86	1.87
Parity	3.38	0.05
Number of children alive	3.20	0.04
Husband’s education level		
No education	20.81	1.23
Primary	43.31	1.45
Secondary/higher	35.88	1.68
Household wealth		
Poorest	21.17	1.26
Poorer	20.41	0.98
Middle	20.49	0.98
Richer	19.37	1.03
Richest	18.57	1.90
Residence		
Rural	89.28	0.89
Urban	10.72	0.89
Region		
Southern region	19.25	1.03
Highland Region	38.96	1.85
Momase region	28.10	1.69
Islands region	13.68	0.81
Adequate antenatal care utilization		
No	48.58	1.57
Yes	51.42	1.57

Concerning the sociodemographic characteristics of women/families, the average age of women was 30.04 years, the average age at first marriage/first cohabitation was 19.55 years, the average number of years of schooling was 5.40 years, the average number of children was approximately 3, the majority (83.47%) of women were unemployed or unpaid, and nearly half (46.86%) had exposure to mass media; the majority (89.28%) of women lived in rural areas, about two fifths (28.10%) were from the Momase region, and 21.17% of women’s households were in the poorest quintile.

### 3.2 EFA and CFA analysis results

**Figure 2:**
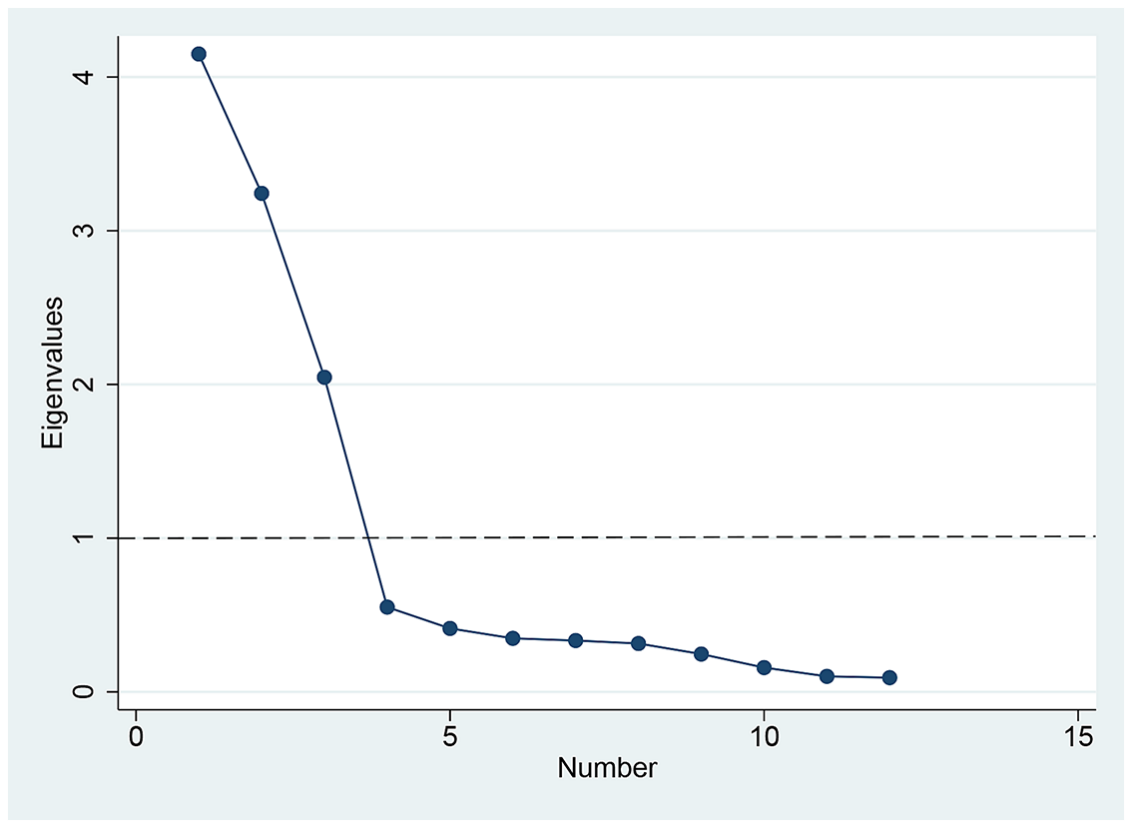
Scree plot of eigenvalues plotted against factors, including 12 variables used for EFA.

The Kaiser-Meyer-Olkin (KMO) test result was 0.77 ( $\chi^{2}$ = 18387.045, *df* = 55, *p* = 0.000), reflecting the applicability of the data to the factor analysis. We split the sample randomly into two independent samples. Half of the sample was used for EFA to explore potential structure (*n* = 2,612), and the other half was used for CFA to test the validity of the structure (*n* = 2,612). The results are as follows:

The EFA section retained three factors based on the Kaiser criterion and the scree plot (see Figure 2), which were rotated to identify three dimensions of women’s empowerment: attitudes toward partner violence, household decision-making, and access to health services, explaining 78.66% of the total variance. The factor loadings for the three-factor structure ranged from 0.690 to 0.954 (see Table 2), and the Cronbach’s alpha coefficient for each domain and overall ranged from 0.739 to 0.842, demonstrating good internal consistency (53).

**Table 2:** Results of factor analysis of women’s empowerment indicators, PNG DHS 2016–2018.

Latent structure	Loading(EFA)	*p*-value (CFA)
Attitudes toward partner violence		
Neglects children	0.884	–
Goes out without telling their husband	0.895	0.000
Argues with husband	0.891	0.000
Refuses to have sex with their husband	0.858	0.000
Burns food	0.791	0.000
Household decision-making		
Women’s health care	0.880	–
Large household purchases	0.783	0.000
Visiting relatives/family	0.895	0.000
Access to health services		
Distance	0.954	–
Money	0.822	0.000
Alone	0.852	0.000
Permission	0.690	0.000

The CFA section indicated the good structural validity of the measurement models based on the significance of the path coefficient (see Table 2) and the model fit index (RMSEA = 0.018, SRMR = 0.047; CFI = 0.991; TLI = 0.988); Mplus limited the path coefficient of the first metric to 1 and therefore did not calculate its statistical significance. The degree of model fit was judged as follows: RMSEA ≤0.05 good fit, ≤ 0.08 acceptable; SRMR ≤0.05 good fit, ≤ 0.1 acceptable; CFI/TLI ≥ 0.97 good fit, ≥ 0.95 acceptable.

### 3.3 SEM analysis results

**Figure 3:**
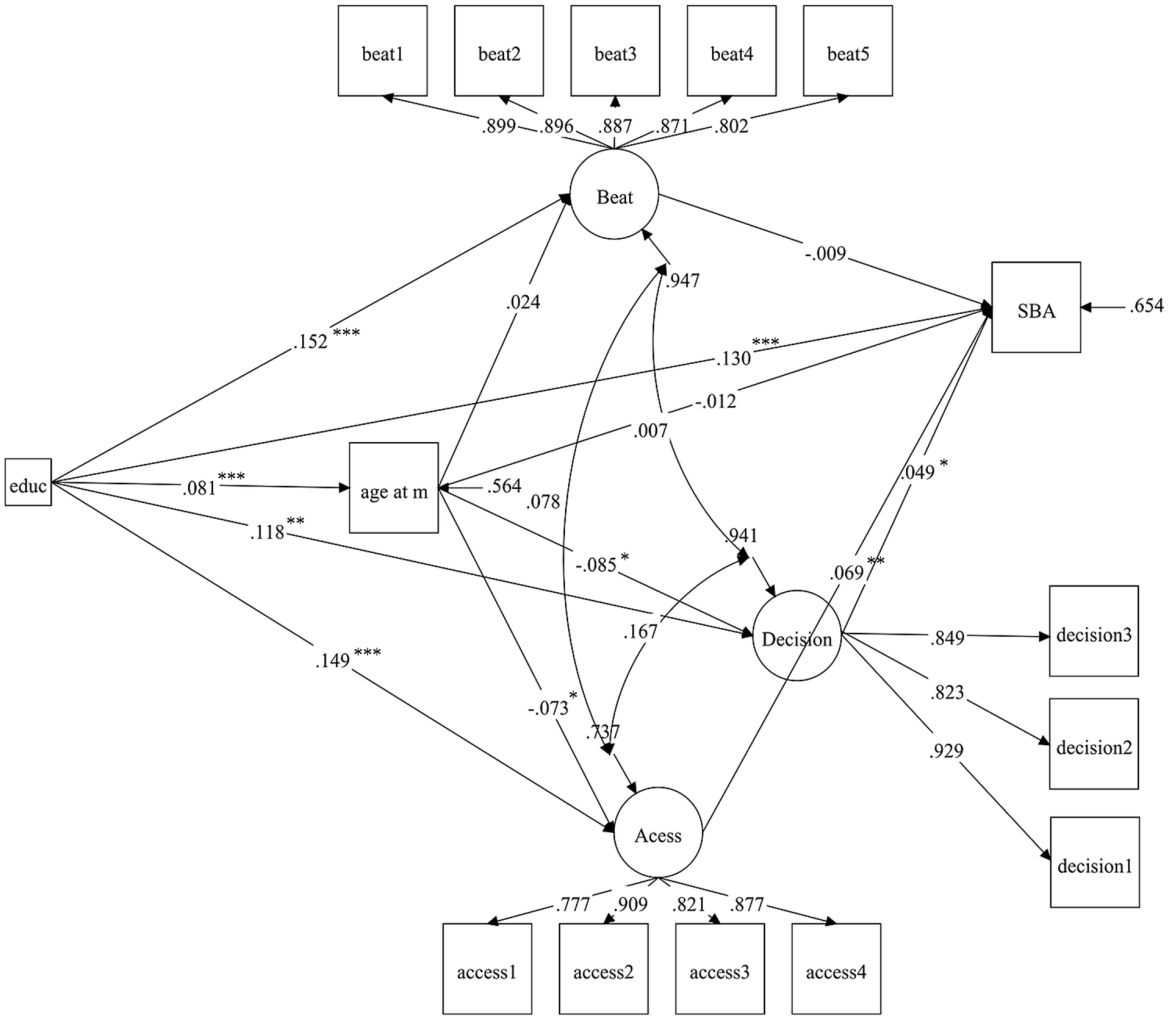
Path diagram for structural equation analysis, PNG DHS 2016–2018, ***p < 0.001, **p < 0.01, *p < 0.05. Beat, attitudes toward partner violence; age at m, age at first marriage; decision, household decision-making; Access, access to health services; SBA, SBA utilization during childbirth. Control variables are not shown in the figure.

The results of the standardized SEM analysis are shown in Table 3 and Figure 3, and the data fitted to the model indicate a good fit (RMSEA = 0.013; SRMR = 0.046; CFI = 0.976; TLI = 0.964).

**Table 3:** Standardized path coefficients for structural equations, PNG DHS 2016–2018.

Predictors in the equation (X)	Dependent variables				
Endogenous variables	Age at first marriage	Attitudes toward partner violence	Household decision-making	Access to health services	SBA utilization during childbirth
Age at first marriage		0.024	−0.085*	−0.073*	−0.012
Household decision-making					0.049*
Attitudes toward partner violence					−0.011
Access to Health services					0.069**
Exogenous variables					
Women’s education level	0.081***	0.152***	0.118**	0.149***	0.130***
Age	0.861***	0.000	0.216***	0.075*	0.037
Working state					
Paid employment	0.034	0.237***	0.153	0.116	0.100*
Exposure to mass media					
Yes	−0.050	−0.091	0.057	0.179**	0.036
Parity	−0.447***	0.077	−0.188	−0.153	−0.156*
Number of children alive	−0.278***	−0.056	0.022	0.146	0.049
Husband’s education level					
Primary	−0.043	−0.217**	0.162*	0.025	0.112*
Secondary/higher	0.030	−0.246**	0.198**	0.130*	0.255***
Household wealth					
Poorer	−0.002	−0.008	−0.159	0.236***	−0.036
Middle	−0.036	0.015	−0.133	0.339***	0.167**
Richer	−0.092	−0.011	−0.106	0.534***	0.296***
Richest	−0.104	0.025	−0.241*	0.827***	0.457***
Residence					
Urban	−0.021	0.107	−0.052	0.273**	0.086
Region					
Highland Region	−0.129***	−0.320***	−0.179*	0.095	0.036
Momase region	0.006	−0.200**	−0.057	−0.008	−0.162**
Islands region	0.168***	−0.134	−0.025	−0.085	0.086
Adequate antenatal care utilization					
Yes					0.501***

***p < 0.001, **p < 0.01, *p < 0.05. Reference groups: working state, unemployed; exposure to mass media, none; husband’s education level, uneducation; household wealth, poorest; residence, rural; region, South region; Adequate antenatal care utilization, none.

Two empowerment variables, household decision-making power (standardized path coefficient, *β* = 0.049, *p* < 0.05) and access to health services (*β* = 0.069, *p* < 0.01), were positively associated with SBA utilization during childbirth; however, the association among attitudes toward partner violence and SBA utilization was statistically insignificant (see Table 3, column 6). Women’s use of SBA was positively associated with higher levels of education, paid employment, adequate use of antenatal services, husband’s primary/secondary education, and higher levels of household wealth, and negatively associated with higher parity and less prevalence in the Highlands than the South. Age at first marriage was positively linked with higher levels of education, older age, adversely linked with higher parity, and more living children; it is lower for women in the Highlands compared to the South and higher for women in the Islands (see Table 3, column 2).

Regarding women’s empowerment (see Table 3, columns 3–5), attitudes toward partner violence are positively correlated with women’s high levels of education and paid employment and negatively correlated with their husbands’ primary/secondary education. Women in the Momase and Highlands regions have more tolerant attitudes toward partner violence than women in the South region. In addition, household decision-making was positively associated with women’s high level of education, older age, and husbands’ primary/secondary education. However, it was negatively associated with older age at first marriage, and women living in the Highlands had more household decision-making power than in the South. Access to health services was positively associated with women’s high level of education, older age, paid work, exposure to mass media, higher level of household wealth, living in the city, husbands’ primary/secondary education,and negatively associated with older age at first marriage.

The bootstrap test results showed that women’s education influenced SBA consumption through multiple pathways (see Table 4), with the standardized total indirect effect being significant (*β* = 0.013, 95% CI: 0.002, 0.026), but only the indirect effect pathway mediated by access to health services was significant (*β* = 0.011, 95% CI: 0.002, 0.022), accounting for 7.69% of the total standardized effect, indicating the importance of access to health services in the influence of women’s educational level on SBA utilization.

**Table 4:** Results of standardized bootstrap mediation test, PNG DHS 2016–2018.

Pathways	β	*p* value	Bootstrapped 95% CI
Total	0.143	0.000	(0.096, 0.185)
Total direct	0.129	0.000	(0.081, 0.174)
Total indirect	0.013	0.029	(0.002, 0.026)
Path of indirect effects			
Age at first marriage	−0.001	0.562	(−0.005, 0.002)
Household decision-making	0.006	0.087	(0.000, 0.014)
Attitudes toward partner violence	−0.002	0.613	(−0.007, 0.004)
Access to health services	0.011	0.029	(0.002, 0.022)
Age at first marriage, then Attitudes toward partner violence	0.000	0.737	(0.000, 0.000)
Age at first marriage, then Household decision-making	0.000	0.203	(−0.001, 0.000)
Age at first marriage, then Access to health services	0.000	0.151	(−0.001, 0.000)

## 4 Discussion

This study investigated the relationship and influential mechanisms among the status of women, women’s empowerment (represented by three latent variables), and SBA utilization. The model encompassing women’s status, empowerment, and SBA utilization in Papua New Guinea was validated through factor analysis. The SEM estimation results indicated that the status and empowerment of women in PNG have significant direct and indirect effects on SBA utilization. Notably, the impact of women’s empowerment on SBA utilization necessitates careful consideration of regional, cultural, and economic contexts.

Drawing on established research and theoretical frameworks (29, 31, 34–36), this study proposed the validated three-factor model of women’s empowerment (decision-making power in the household, attitudes toward violence, and access to healthcare), confirming the viability of employing multiple dimensions to measure women’s empowerment at the individual level in PNG. Additionally, the outcomes of the factor analysis endorse the adoption of a more extensive set of empowerment indicators, facilitating a more comprehensive assessment of women’s empowerment.

The study found a significant influence of education on the age at first marriage, women’s empowerment (across three dimensions), and SBA utilization, aligning with findings in related studies within developing countries. Improved education levels have the potential to prevent early marriage and detrimental marital predispositions (42). However, caution is warranted in exploring the connection between education and age at first marriage due to potential bidirectionality (55). Meanwhile, education manifested a favorable impact on economic autonomy (56), with higher educational attainment correlating with increased economic independence. Women endowed with economic autonomy experience heightened financial security and assertive participation in family decision-making (57), thereby diminishing reliance on potentially abusive partners (58). Moreover, education serves as a facilitator for maternal learning, fostering awareness of appropriate maternal and child health services and rectifying improper practices (e.g., solitary or inadequately supported childbirth) and attitudes (30, 59). Furthermore, education is a positive factor in maternal learning, raising awareness of appropriate maternal and child health services, and correcting inappropriate practices and attitudes (30). These findings underscore the pivotal role of education in advancing both women’s empowerment and women’s health.

The study also supported the robust correlation between the two dimensions of women’s empowerment and SBA utilization in PNG. The extent of women’s participation in household decision-making varies in different regions of PNG. For instance, women’s livelihoods in Chimbu and Jiwaka are usually determined by their partners or male relatives (60). Advocating for women’s participation in household decision-making may prevent women from losing autonomy over their health (61), especially in poor households, where spending on health services often constitutes a significant portion of the budget (62). Enhanced accessibility to health services mitigates women’s constraints related to income, geographical marginalization, transportation barriers, and safety concerns outside the home when seeking SBA services. This, in turn, contributes to reducing delays in women’s utilization of SBA (63). These findings underscore the imperative for governments and policymakers to prioritize comprehensive women’s empowerment and emphasize key dimensions to enhance women’s empowerment and ensure women’s health and well-being.

Our investigation revealed a synergistic impact between education and women’s empowerment on SBA utilization. Increases in education and women’s empowerment may contribute to accelerating women’s utilization of SBA. Correspondingly, research from Tanzania also showed that women’s empowerment plays a moderating role between education and SBA utilization, but the dimensions at play are not the same (29). This divergence may stem from the influence of contextual factors such as geography, religion, and culture on women’s empowerment (64). Therefore, the design of empowerment programs should be tailored to regional or national realities, aiming to concurrently enhance women’s empowerment, eradicate gender inequality, and ameliorate maternal health outcomes.

In PNG, the effect of age at first marriage on SBA utilization is not significant. Although partial evidence from developing countries (65, 66) and the social context within PNG enables the formulation of hypotheses, the intricate nature of women’s behavior in accessing health services (63, 67), particularly in resource-poor settings (68), implies that unobserved mediators may be present in the relationship between age at first marriage and SBA utilization. Furthermore, SEM allows for a correlation between the residual terms of women’s empowerment. The SEM results indicate the presence of interconnected unobserved components within the dimensions of women’s empowerment, necessitating additional research for elucidation.

As the first theoretically based study in PNG to examine the complex mechanisms of SBA utilization in the context of women’s status and empowerment at childbirth using nationally representative data, it could serve as a standard and impetus for future research on similar topics in PNG and other Pacific Island countries. Simultaneously, the application of SEM provides this study with several advantages, such as effectively controlling measurement error and allowing the examination of causal associations between variables. However, there are still some limitations to this study. First, the study sample’s representativeness and the results’ generalizability are more limited because unmarried women were not included. Second, because the empowerment dimensions are limited to DHS data, the women’s empowerment studies cannot have all dimensions that may be examined. Third, despite the advantages of SEM in causal inference, the certainty of causal associations is still limited and must be validated through longitudinal studies based on long-term data.

## 5 Conclusion

Based on PNG DHS 2016–2018 cross-sectional data, this study validated the mechanism of action between women’s status, empowerment, and SBA utilization during childbirth. The findings indicated the multidimensional nature of women’s empowerment and validated the direct and indirect impacts of women’s status and empowerment on using SBA during childbirth. This evidence suggests further empirical research-based interventions in PNG and possibly PSIDS to improve women’s education, household decision-making power, and access to health services through a joint effort at the individual, community, and societal levels to promote women’s SBA utilization during childbirth, to improve maternal and neonatal health and well-being in PNG, and to achieve the SGD 3.1 target.

## Data availability statement

The datasets generated and analyzed during this study are available in the DHS program repository. This data can be found here: [https://dhsprogram.com/data/dataset/Papua-New-Guinea_Standard-DHS_2017.cfm?flag=1].

## Ethics statement

The studies involving humans were approved by Inner City Fund International Institutional Review Board. The studies were conducted in accordance with the local legislation and institutional requirements. The participants provided their written informed consent to participate in this study.

## Author contributions

HS contributed to conceptualization, formal analysis, and writing – original draft. HZ contributed to the writing – original draft, review, and revision of the article. BW was responsible for literature management, resources, visualizations, and writing – review and editing. YJ contributed to supervision, reviews, and project management. All authors read and approved the content of the final manuscript.
